# Stereotactic Body Radiotherapy for Oligometastatic Disease in Melanoma Patients Receiving Immunotherapy: A Single-Center Retrospective Analysis

**DOI:** 10.3390/cancers18111812

**Published:** 2026-06-01

**Authors:** Matea Lekić, Hrvoje Kaučić, Domagoj Kosmina, Ivan Prološčić, Giovanni Ursi, Vanda Leipold, Sunčana Divošević, Maja Karaman Ilić, Karla Schwarz, Dragan Schwarz, Damir Vučinić

**Affiliations:** 1 Specialty Hospital Radiochirurgia Zagreb, 10431 Sveta Nedelja, Croatia; matea.lekic@radiochirurgia.hr (M.L.); hrvoje.kaucic@radiochirurgia.hr (H.K.); domagoj.kosmina@radiochirurgia.hr (D.K.); ivan.proloscic@radiochirurgia.hr (I.P.); giovanni.ursi@radiochirurgia.hr (G.U.); vanda.leipold@radiochirurgia.hr (V.L.); suncana.divosevic@radiochirurgia.hr (S.D.); maja.karaman.ilic@radiochirurgia.hr (M.K.I.); karla.schwarz@radiochirurgia.hr (K.S.); dragan.schwarz@radiochirurgia.hr (D.S.); 2 Department of Physics, Faculty of Science, University of Zagreb, 10000 Zagreb, Croatia; 3 Medical Faculty Osijek, Josip Juraj Strossmayer University of Osijek, 31000 Osijek, Croatia; 4 Faculty of Health Studies, University of Rijeka, 51000 Rijeka, Croatia; 5 Faculty of Dental Medicine and Health, Josip Juraj Strossmayer University of Osijek, 31000 Osijek, Croatia; 6 Medical Faculty, Juraj Dobrila University of Pula, 52100 Pula, Croatia

**Keywords:** immune checkpoint inhibitors, immunotherapy, melanoma, oligometastatic disease, radioimmunotherapy, stereotactic body radiotherapy

## Abstract

Background/Objectives: Metastatic melanoma increasingly includes clinical scenarios in which metastasis-directed treatment may complement systemic immunotherapy, particularly in oligometastatic disease. We evaluated outcomes of stereotactic body radiotherapy (SBRT) combined with immunotherapy in patients with metastatic melanoma, focusing on local control, survival, treatment sequencing, and safety. Methods: In this retrospective single-center study, 63 patients underwent SBRT for 97 extracranial metastatic lesions while receiving immune checkpoint inhibitors. The primary endpoint was local control (LC), while secondary endpoints included progression-free survival (PFS), overall survival (OS), and treatment-related toxicity. Results: LC rates at 12, 24, and 36 months were 95.9%, 89.7%, and 89.7%, respectively, demonstrating durable long-term control of treated lesions. Median OS was 47 months, while median PFS was not reached. A numerical trend toward longer PFS was observed among patients receiving SBRT during immunotherapy, although the differences were not statistically significant. No significant differences in LC were identified across oligometastatic disease subcategories. Combined treatment was well tolerated, with predominantly low-grade toxicity and no signal of increased immune-related adverse events. Conclusions: SBRT combined with immunotherapy appears to be a feasible and effective metastasis-directed treatment strategy in selected patients with metastatic melanoma. Durable local control, encouraging survival outcomes, and favorable tolerability support further prospective studies to optimize treatment sequencing and the integration of local and systemic therapies.

## Simple Summary

Melanoma patients with a limited number of metastases are increasingly treated with immunotherapy, but some tumors may continue to grow despite otherwise effective systemic treatment. In this study, we evaluated stereotactic body radiotherapy, a highly precise form of radiation treatment, combined with immunotherapy in 63 patients with metastatic melanoma. The treatment achieved excellent long-term local tumor control, with nearly 90% of treated metastases remaining controlled three years after radiotherapy. Median overall survival reached 47 months. Treatment was well tolerated, with very few severe side effects. Patients receiving radiotherapy before or during immunotherapy showed numerically better outcomes, supporting the concept that radiotherapy may enhance antitumor immune responses. Our findings highlight the potential of combining stereotactic radiotherapy with immunotherapy as an effective treatment strategy for selected patients with oligometastatic melanoma.

## 1. Introduction

Metastatic melanoma (MM) has shifted from being a uniformly fatal disease to one where long-term survival and, in some cases, lasting remission are now possible. However, this progress has come at the cost of increasing biological and clinical complexity, with highly heterogeneous patterns of response and progression emerging in the era of modern systemic therapy [1]. Despite remarkable advances in immune checkpoint inhibitors, a substantial proportion of patients experience primary resistance or develop limited, spatially heterogeneous progression while receiving otherwise effective treatment. These clinical scenarios challenge traditional treatment paradigms and demand more nuanced, multidisciplinary approaches that integrate systemic and local therapies [2,3].

Over the past decade, the therapeutic landscape of advanced melanoma has undergone a paradigm shift driven by the introduction of immune checkpoint inhibitors (ICIs). Agents targeting cytotoxic T-lymphocyte-associated antigen 4 (CTLA-4) and programmed cell death protein 1 (PD-1) have fundamentally altered the natural history of the disease, enabling durable responses and long-term survival in a subset of patients. Early phase III data demonstrated that nivolumab significantly improved overall survival (OS), progression-free survival (PFS), and response rates compared with chemotherapy in treatment-naïve patients [4]. These findings were further supported by long-term follow-up data from KEYNOTE-006 trial, in which pembrolizumab demonstrated a sustained survival benefit over ipilimumab, with a 10-year OS rate of 34.0%, highlighting the potential for long-term disease control [5]. Combination strategies have further improved outcomes. In particular, dual checkpoint blockade with nivolumab plus ipilimumab has emerged as one of the most effective first-line approaches in MM, achieving high response rates and unprecedented long-term survival, as demonstrated in the CheckMate 067 trial with 10-year follow-up [6]. Despite these advances, a substantial proportion of patients either fail to respond or eventually develop acquired resistance. This clinical heterogeneity reflects the complex biology of melanoma and underscores the need for complementary treatment strategies.

SBRT has emerged as a highly effective local treatment modality in this setting, providing excellent two-year local control rates exceeding 90% with minimal toxicity [7]. Importantly, emerging evidence suggests that treating all metastatic sites may improve PFS and OS, supporting the concept of metastasis-directed therapy in selected patients. This pattern reflects spatially heterogeneous resistance and presents a therapeutic opportunity: local treatment of resistant clones may allow continuation of effective systemic therapy, thereby prolonging disease control without immediate treatment escalation [8,9,10]. In daily clinical practice, these scenarios are becoming increasingly common in the era of immunotherapy, yet optimal management strategies remain poorly defined. In particular, the integration of SBRT with ICIs, optimal patient selection, and the timing of local interventions relative to systemic therapy remain subjects of ongoing debate.

Building on this evolving therapeutic landscape, our study focuses on a real-world cohort of patients with MM treated with SBRT in combination with contemporary ICIs. Considering the heterogeneity observed in progression patterns in our dataset, including oligometastatic, oligoprogressive, and oligopersistent disease, we aim to provide clinically relevant insights into outcomes following metastasis-directed therapy. Specifically, the objective of this study is to evaluate local control, PFS, and OS in patients with metastatic melanoma undergoing SBRT in conjunction with immunotherapy, and to explore the impact of disease state and treatment sequencing.

## 2. Materials and Methods

### 2.1. Study Design and Patient Population

This retrospective single-center cohort study was conducted at the Specialty Hospital Radiochirurgia Zagreb, Croatia, and included consecutive patients treated between January 2018 and December 2023. Eligible patients were aged ≥18 years, had histologically confirmed melanoma with oligometastatic disease, underwent SBRT to at least one extracranial metastatic lesion, and had complete clinical, treatment, and follow-up data available for analysis. Patients with controlled intracranial disease were not excluded. All patients had an Eastern Cooperative Oncology Group (ECOG) performance status ≤2 and an estimated life expectancy of at least six months at the time of SBRT.

Metastatic disease was established using standard radiological imaging, including computed tomography (CT), magnetic resonance imaging (MRI), and/or positron emission tomography (PET/CT). Oligometastatic disease was defined in accordance with contemporary clinical practice as a limited metastatic burden amenable to metastasis-directed ablative treatment and was further classified, according to ESTRO-EORTC recommendations, as oligoprogression, oligopersistence, or oligorecurrence [11]. Oligorecurrence was defined as limited metastatic relapse during a treatment-free interval, oligoprogression as progression in a limited number of lesions during ongoing systemic therapy, and oligopersistence as residual stable or partially responding disease persisting during or following systemic treatment.

ICI regimens were administered according to standard melanoma dosing schedules and institutional clinical practice. Pembrolizumab was given at a dose of 200 mg every 3 weeks or 400 mg every 6 weeks. Nivolumab was administered at 240 mg every 2 weeks or 480 mg every 4 weeks. Combination immunotherapy consisted of nivolumab 1 mg/kg plus ipilimumab 3 mg/kg every 3 weeks for four doses, followed by nivolumab maintenance. Treatment was continued until disease progression, unacceptable toxicity, or completion of the planned treatment course.

### 2.2. SBRT Technique and Systemic Therapy

Patients were treated with either the Varian EDGE linear accelerator or the CyberKnife S7 robotic radiosurgery system, per institutional protocols. Simulation, treatment planning, and delivery were individualized based on lesion site and motion characteristics. Target delineation relied on multimodal imaging, including diagnostic CT, MRI, and PET/CT, where available. Motion management strategies, including image guidance, respiratory motion management, and fiducial or real-time tracking when indicated, were implemented based on lesion location and treatment platform. SBRT was delivered with ablative-intent dose-fractionation schedules selected according to lesion size, anatomic location, prior treatments, and organ-at-risk constraints. Both single- and multiple-hypofractionated sessions were used. Treatments were delivered with stereotactic image guidance using high-precision platforms, including high-dose-rate delivery where applicable. All patients received systemic therapy with ICIs, administered before, during, or after SBRT, based on multidisciplinary clinical decision-making. Based on temporal sequencing, patients were categorized into three groups according to whether SBRT was delivered before, during, or after the initiation of immunotherapy.

### 2.3. Endpoints and Follow-Up

The primary endpoint was LC, defined as the time from SBRT initiation to radiologically confirmed progression within the treated lesion. Secondary endpoints included PFS, OS, and treatment-related toxicity. PFS was calculated from the start of ICIs to disease progression or death from any cause, while OS was calculated from ICI initiation to death from any cause. Follow-up data were obtained from clinical records and serial imaging assessments. For local control analysis, each treated lesion was considered at risk from the start of SBRT until radiologically confirmed in-field progression; lesions without local progression were censored at the date of last imaging follow-up or death. Treatment-related toxicity was assessed retrospectively using the Common Terminology Criteria for Adverse Events (CTCAE), with specific evaluation of both SBRT-related toxicity and immune-related adverse events occurring following SBRT.

### 2.4. Statistical Analysis

Statistical analysis was performed using IBM SPSS Statistics version 27.0. Continuous variables were presented using measures of central tendency and dispersion, including mean, standard deviation, median, and interquartile range, depending on data distribution. The normality of distribution was assessed using the Kolmogorov–Smirnov test. Comparisons between groups for continuous variables were performed using the Mann–Whitney U test. Associations between categorical variables were analyzed using Fisher’s exact test. Survival outcomes, including PFS and OS, were evaluated using the Kaplan–Meier method, with comparisons performed using the log-rank test. A *p*-value < 0.05 was considered statistically significant.

## 3. Results

A total of 63 patients with MM treated with a combination of SBRT and immunotherapy were included in this analysis. Baseline clinicopathological and treatment characteristics are summarized in Table 1. Additional subgroup analyses were performed according to histological subtype, BRAF status, and type of immunotherapy. Among patients with superficial spreading melanoma (n = 32) and nodular melanoma (n = 31), no statistically significant differences were observed in either PFS (log-rank χ^2^ = 0.687, *p* = 0.407) or OS (log-rank χ^2^ = 0.469, *p* = 0.493). Similarly, survival outcomes did not differ significantly between patients with BRAF-mutant disease (n = 21) and BRAF wild-type disease (n = 42), for either PFS (log-rank χ^2^ = 1.004, *p* = 0.316) or OS (log-rank χ^2^ = 0.351, *p* = 0.554). Regarding the immunotherapy regimen, 46 patients received pembrolizumab, 15 received nivolumab, and 2 received ipilimumab plus nivolumab. Because of the markedly uneven distribution of patients across immunotherapy subgroups, particularly the small number receiving combination immunotherapy, comparisons according to immunotherapy regimen were considered descriptive.

At initial diagnosis, the largest proportion of patients presented with stage IV disease (34.9%), with the remainder distributed across earlier AJCC stages. At the time of SBRT indication, most lesions were classified as oligopersistent (47.6%) or oligoprogressive (44.4%), whereas oligorecurrent disease was less common (7.9%), indicating that SBRT was predominantly applied in the context of residual or progressive disease under systemic therapy.

All patients received combined treatment with immunotherapy and SBRT. Pembrolizumab was the most frequently administered agent (73.0%), followed by nivolumab (23.8%), while combination therapy with ipilimumab and nivolumab was used in a minority of cases (3.2%). Regarding treatment sequencing, SBRT was most commonly delivered concurrently with immunotherapy (50.8%), followed by administration after immunotherapy (39.7%), and less frequently prior to systemic treatment initiation (9.5%).

A total of 97 metastatic lesions were treated with SBRT. Most patients had a limited number of treated lesions (1–5), and in cases with multiple metastases, all lesions were treated within a single SBRT course. The most frequent sites of treated lesions were lymph nodes (39.2%) and lungs (37.1%), followed by the liver (14.4%), bone (5.2%), adrenal glands, and other less common locations, reflecting a predominance of nodal and pulmonary oligometastatic disease.

The mean total radiation dose was 33.2 ± 5.1 Gy (range, 20–45 Gy), and the mean number of fractions was 2.57 ± 1.66 (range, 1–5). Nearly half of all lesions (47.4%) were treated with a single-fraction regimen, indicating a frequent use of ablative SBRT schedules. The median follow-up duration was 45.5 months, allowing for a robust assessment of long-term outcomes.

**Figure 1:**
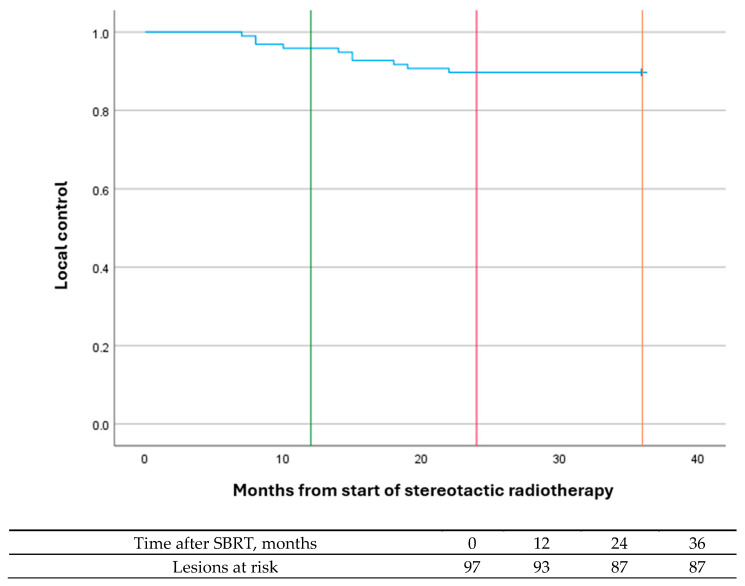
Kaplan–Meier curve of local control following stereotactic body radiotherapy (SBRT) in patients with metastatic melanoma receiving immunotherapy. Durable local control was observed, with rates of 95.9%, 89.7%, and 89.7% at 12, 24, and 36 months, respectively, demonstrating sustained long-term control of treated lesions. Vertical lines indicate the 12-, 24-, and 36-month time points at which local control rates were assessed, marked in green, red, and orange, respectively.

Local control rates were high and sustained over time. At 12 months following SBRT, 95.9% of lesions remained locally controlled, while local control at 24 and 36 months was 89.7%, indicating durable treatment efficacy with minimal late local failures (Figure 1).

**Figure 2:**
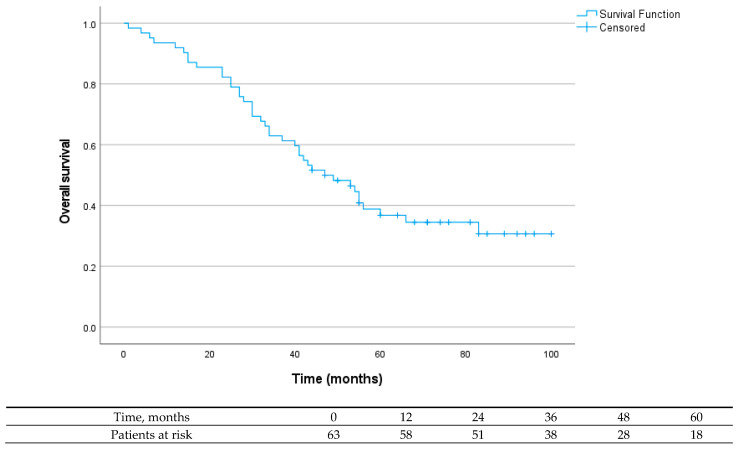
Kaplan–Meier analysis of overall survival in patients with metastatic melanoma treated with SBRT and immunotherapy. Median overall survival was 47 months, demonstrating encouraging long-term survival outcomes in this oligometastatic cohort.

**Figure 3:**
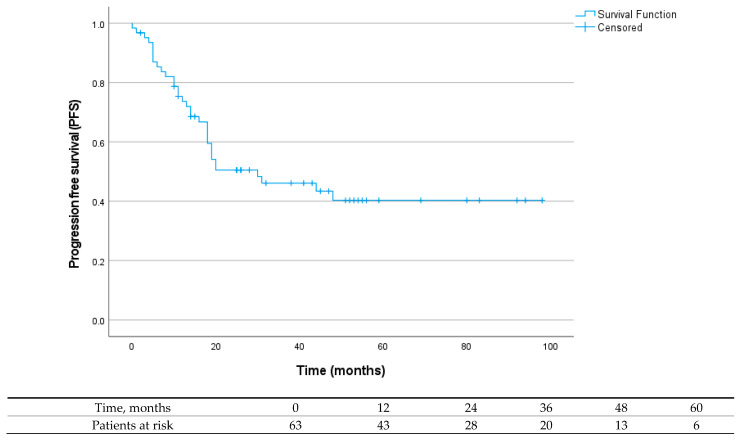
Kaplan–Meier analysis of progression-free survival in patients with metastatic melanoma treated with combined stereotactic body radiotherapy and immunotherapy, demonstrating durable disease control, with median progression-free survival not reached.

Survival outcomes were also favorable. The mean OS was 55.92 months (95% CI, 47.34–64.49), with a median OS of 47 months (95% CI, 33.35–60.64), indicating prolonged survival in this oligometastatic population treated with combined-modality therapy (Figure 2). The mean PFS was 33.69 months (95% CI, 32.30–35.08), with median PFS not reached, suggesting that more than half of the patients remained progression-free at three years (Figure 3).

No statistically significant difference in total radiation dose was observed between lesions with and without local progression (Mann–Whitney U test, U = 311, *p* = 0.122). However, lesions with local progression received a numerically lower mean dose than locally controlled lesions (30.4 Gy vs. 33.5 Gy), although this difference was not statistically significant and should be interpreted descriptively only. Given the small number of local progression events, lesion heterogeneity, anatomical site differences, and individualized dose selection based on organ-at-risk constraints, these data do not establish a dose–response relationship.

Analysis of fractionation did not demonstrate a statistically significant association with treatment outcomes. Specifically, no significant relationship was found between the number of fractions and local control (Fisher’s exact test, χ^2^ = 2.112, *p* = 0.319), nor between fractionation and disease progression (χ^2^ = 2.084, *p* = 0.322). Local progression events were observed across all fractionation regimens, without a clear dose–fractionation pattern. Importantly, the highest proportion of local control loss was observed in lesions treated with single-fraction SABR, accounting for 8.5% of cases (n = 7). However, given the limited number of progression events and the relatively small subgroup sizes, this finding should be interpreted with caution and does not indicate a definitive disadvantage of single-fraction treatment.

**Figure 4:**
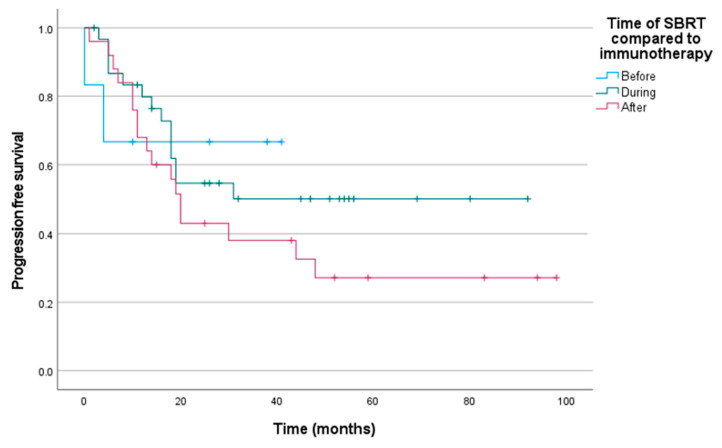
Kaplan–Meier analysis of progression-free survival according to the timing of stereotactic body radiotherapy relative to immunotherapy (before, during, or after immunotherapy). A numerical trend toward improved progression-free survival was observed in patients treated with SBRT during immunotherapy, although differences did not reach statistical significance.

When stratified according to the timing of SBRT relative to immunotherapy, numerical differences in PFS were observed. Patients treated with SBRT prior to immunotherapy had a median PFS that was not reached, suggesting durable disease control in this subgroup. Similarly, median PFS was not reached in patients receiving SBRT during immunotherapy, indicating the most favorable progression-free outcomes. In contrast, patients treated with SBRT after immunotherapy had a median PFS of 20 months (95% CI, 16.93–23.07). Although patients treated before or during immunotherapy appeared to have a longer PFS than those treated after immunotherapy, these differences did not reach statistical significance, likely due to the limited sample size. The subgroup sizes were limited and uneven, with 6 patients treated before immunotherapy, 32 during immunotherapy, and 25 after immunotherapy, which restricts the statistical power and precludes definitive conclusions regarding optimal treatment sequencing (Figure 4).

**Figure 5:**
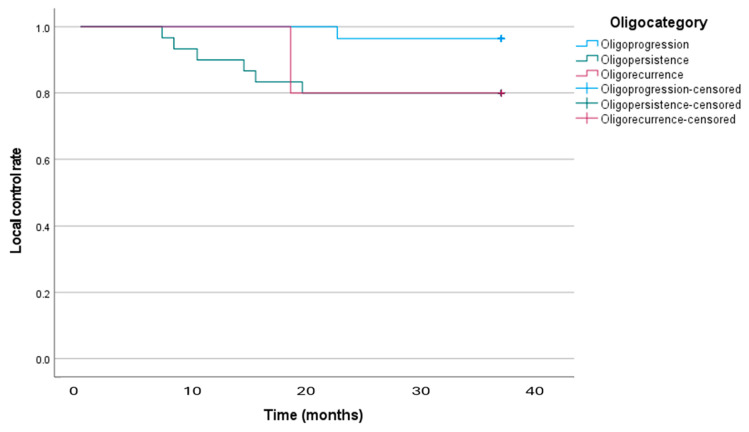
Kaplan–Meier analysis of local control by oligometastatic disease category (oligoprogression, oligopersistence, and oligorecurrence). No significant differences in local control were observed across oligometastatic subgroups, supporting comparable efficacy of SBRT across oligometastatic states.

Further analysis according to ESTRO-EORTC oligometastatic disease categories showed no statistically significant difference in local control among patients with oligoprogression, oligopersistence, and oligorecurrence (log-rank χ^2^ = 3.86, *p* = 0.145). The subgroup distribution was unbalanced, with 28 patients classified as oligoprogressive, 30 as oligopersistent, and 5 as oligorecurrent. Mean local control was 35.50 months for oligoprogression (95% CI, 34.54–36.46), 31.23 months for oligopersistence (95% CI, 27.76–34.71), and 32.40 months for oligorecurrence (95% CI, 26.09–38.71). Local control remained high across all subgroups, with numerically highest rates observed in patients with oligoprogressive disease, although these differences did not reach statistical significance (Figure 5).

Treatment was generally well tolerated. SBRT-related toxicity was predominantly low-grade, with grade 1 events observed in 12.9% of patients and grade 2 events in 6.4%. Only one patient (1.6%) experienced grade 3 toxicity, presenting with rectal bleeding following SBRT of a pelvic lesion. The patient who experienced this event was receiving immunotherapy during SBRT. Immunotherapy-related adverse events occurring after SBRT were infrequent (12%) and were mostly low-grade, indicating no apparent increase in toxicity associated with combined treatment (Table 2).

## 4. Discussion

A principal finding of our study is the durable, high rate of local control after SBRT in patients with MM, all of whom were uniformly treated with immunotherapy. The local control rates at 12, 24, and 36 months were 95.9%, 89.7%, and 89.7%, respectively. These findings support SBRT as an effective metastasis-directed treatment strategy in melanoma, despite the disease’s historically perceived radioresistance. Notably, lesion control remains durable beyond two years, indicating that in carefully selected patients receiving modern systemic therapy, SBRT can offer sustained control of treated lesions. Our results compare favorably with existing melanoma- specific SBRT series. For instance, in a recent multicenter French study by Trentesaux et al., local control rates were 94.2%, 90.3%, and 90. 3% at 1, 2, and 3 years, closely aligning with our findings [7]. Similarly, Franceschini et al. reported excellent long- term local control following extracranial SBRT in oligometastatic melanoma, supporting the consistency of durable lesion control across institutions [12]. Earlier studies by Stinauer et al. also demonstrated high lesion control rates despite smaller and more heterogeneous cohorts [13]. Collectively, these studies underscore the reproducibility of high LC rates with SBRT in melanoma. Significantly, variations among the studies should be interpreted in light of substantial differences in patient selection and exposure to systemic therapy. Unlike several previous retrospective series in which immunotherapy was absent, inconsistently administered, or given only to a subset of patients, all patients in our cohort received ICIs, a clinically significant distinction. This is particularly relevant as emerging evidence suggests that LC cannot be interpreted independently of the systemic immune environment in which SBRT is delivered. For example, in the cohort reported by Backlund et al., the combination of immunotherapy with radiotherapy was associated with improved responses in irradiated lesions compared to radiotherapy alone, supporting the hypothesis that immunotherapy may enhance local treatment efficacy [14]. Similarly, the randomized phase II CHEERS trial further reinforced the rationale for combining ICIs with SBRT, demonstrating improved PFS with combined therapy in patients with advanced solid tumors [15].

In our series, median overall survival of 47 months and prolonged PFS compare favorably not only with melanoma-directed SBRT series, but also with outcomes reported in pivotal immunotherapy trials, despite the fundamentally different study populations. In the cohort by Trentesaux et al., median PFS and OS were similarly encouraging, supporting the consistency of the survival benefit observed in oligometastatic melanoma treated with ablative radiotherapy [7]. By contrast, in KEYNOTE-006 trial, pembrolizumab achieved a median OS of 32.7 months and median PFS of 9.4 months, while the 10-year overall survival rate reached 34%, establishing a benchmark for long-term survival with anti–PD-1 monotherapy [1,5]. Likewise, CheckMate 067 demonstrated unprecedented long-term survival with the combination of nivolumab and ipilimumab, further redefining expectations in advanced melanoma [6]. Although direct comparison between our results and these phase III trials should be made cautiously, the favorable outcomes in our cohort likely reflect several factors, including lower disease burden inherent to oligometastatic disease, favorable patient selection, and potentially the additional contribution of SBRT as a metastasis-directed strategy beyond systemic therapy alone [3,11]. Indeed, one plausible explanation for numerically favorable outcomes in our study compared with broader immunotherapy trials lies in the distinct biology of oligometastatic disease itself. Unlike KEYNOTE-006 and CheckMate 067 trials, which enrolled unselected MM populations, including patients with extensive metastatic burden, our cohort represents a biologically selected subgroup in whom limited disease extent may confer increased susceptibility to both durable immune-mediated control and consolidation with ablative local therapy. In that context, SBRT may contribute not only by eradicating resistant lesions but also through synergy with PD-1 blockade, a concept supported by both translational evidence and prospective data. The randomized phase II CHEERS trial further supports the clinical feasibility and rationale of combining SBRT with immune checkpoint inhibition in advanced solid tumors, although it did not establish a definitive OS benefit and should not be interpreted as direct evidence of benefit in melanoma-specific oligometastatic disease. Therefore, our findings should be interpreted as hypothesis-generating and supportive of prospective, ideally multicenter, controlled studies designed to clarify the independent contribution of SBRT, the optimal timing of treatment, and patient selection criteria. The heterogeneity of immunotherapy regimens, irradiated anatomical sites, and SBRT dose-fractionation schedules constitutes an additional limitation and exemplifies real-world multidisciplinary practice. In the context of metastatic melanoma, the selection of ICIs is not dictated by the anatomical site designated for SBRT but is instead guided by established treatment indications, previous therapies, patient characteristics, comorbidities, BRAF mutation status, toxicity considerations, and multidisciplinary decision-making. Interestingly, despite applying the ESTRO-EORTC oligometastatic classification, we did not observe differences in LC, PFS, or OS across oligorecurrence, oligopersistence, and oligoprogression [11,15]. While one might hypothesize a prognostic gradient among these states, our findings suggest that in immunotherapy-treated melanoma, such distinctions may be attenuated. One possible explanation is that under PD-1 blockade, biological responsiveness to immune therapy may outweigh the prognostic impact of the oligometastatic state itself. In melanoma, oligoprogression may not necessarily represent globally resistant disease, but rather focal immune escape amenable to salvage with local ablative treatment while systemic immune control is maintained. This interpretation is supported by emerging oligometastatic literature, including the melanoma analysis by Heurlin et al., in which survival differences across oligometastatic subcategories were likewise not clearly demonstrated, suggesting that disease state subclassification may have less prognostic relevance when patients are selected for metastasis-directed treatment [16]. In the contemporary immunotherapy era, our data suggest that effective local control with SBRT can be achieved across different oligometastatic subcategories; however, whether this translates into improved survival remains uncertain and cannot be determined without a control group.

Beyond survival outcomes, our findings raise an important biological question, particularly given that patients receiving SBRT before or during immunotherapy showed numerically longer PFS. A plausible explanation lies in the increasingly recognized synergy between ablative radiotherapy and ICIs. Rather than acting solely through direct cytotoxicity, SBRT may function as an immune-modulating intervention capable of amplifying antitumor immunity, a concept particularly relevant in melanoma given its intrinsic immunogenicity and the recognized prognostic role of tumor-infiltrating lymphocytes (TILs) [17,18]. This concept may also be supported by our previous observations using the modified BRISK classification, in which BRISK B tumors, characterized by continuous TILs, showed the highest MITF expression and were associated with increased PD-L1 expression, suggesting a biologically inflamed yet immune-regulated tumor phenotype [2,19]. Such tumors may represent precisely the microenvironment in which SBRT-induced antigen release and immune priming can be most effectively amplified by PD-1 blockade.

This interaction can be viewed through the lens of the emerging “6th R” of radiobiology—reactivation of the immune response. Beyond the classical 5Rs, tumor control after SBRT may also depend in part on immune-mediated elimination of damaged tumor cells. Radiation-induced immunogenic cell death, through calreticulin exposure, ATP and HMGB1 release, dendritic-cell priming, and CD8+ T-cell activation, can transform irradiated lesions into an in situ vaccine [20,21]. This may be especially relevant in melanoma, where pre-existing TIL-rich microenvironments could potentiate radiation-driven immune amplification.

At the microenvironmental level, this synergy is likely mediated through cGAS-STING signaling, type I interferon induction, enhanced antigen presentation, and increased T-cell trafficking via radiation-induced chemokine signaling [22]. SBRT can also influence macrophage polarization and the balance of inflammatory cytokines within the tumor microenvironment [17]. At the same time, because radiation may induce adaptive immune resistance, including PD-L1 upregulation or recruitment of suppressive myeloid populations, these effects may be counterbalanced by concurrent checkpoint blockade, providing a biologically plausible explanation for the favorable outcomes observed with SBRT delivered before or during immunotherapy [23].

This interpretation is also supported by sequencing data. Colangelo et al. showed that the sequence of PD-1 blockade relative to irradiation critically determines induction of systemic immune responses, while Vanpouille-Box et al. demonstrated that fractionated radiation below the TREX1 induction threshold preserves cGAS-STING signaling and optimizes interferon-driven immune priming. These observations may help explain why, in our cohort, treatment timing appeared not merely technical but biologically relevant [21,22].

These mechanistic considerations also raise the question of whether radiation dose and fractionation may influence not only direct tumor control but also the efficacy of combined radioimmunotherapy itself. In our cohort, the largest proportion of local failures occurred after single-fraction SBRT, which is intriguing given the conventional assumption that single high-dose treatments are maximally ablative. Although numbers are limited, this may suggest that in the setting of immunotherapy, purely ablative dosing may not always be biologically optimal. One possible explanation comes from experimental observations that very high single-fraction doses may induce TREX1-mediated degradation of cytosolic DNA, attenuating cGAS-STING signaling and reducing type I interferon–driven immune priming, whereas moderately hypofractionated regimens may better preserve immunogenic signaling [21,22,24]. Similarly, the trend in our data suggesting greater risk of local progression in lesions treated with lower mean dose supports a possible dose–response relationship, although this should not be interpreted as evidence that dose de-escalation can be routinely adopted in patients receiving immunotherapy. Rather, it may suggest that dose selection in this setting depends not only on the ablative effect per lesion but also on whether all visible disease is comprehensively treated while active systemic immune control is maintained. Dose also cannot be considered independently from radiation exposure to the immune system itself. Jin et al. proposed the immune system as an organ at risk, showing that a higher effective dose to circulating immune cells was associated with worse tumor control and survival [25]. This is particularly relevant when considering dose rate, an often underappreciated parameter in SBRT. Higher dose rates may reduce the exposure time of circulating lymphocytes within the radiation field and potentially facilitate lymphocyte preservation, a concept supported by radiobiological modeling indicating increased lymphocyte survival with higher dose rates [26]. In parallel, Herrera et al. have suggested that dose rate may influence not only lymphocyte sparing but also broader immunological responsiveness, adding another layer to optimization of SBRT–ICIs combinations [27]. More broadly, the clinical rationale for combining radiotherapy with immune checkpoint inhibition is also supported by emerging experience in other immunogenic skin cancers. For example, in advanced cutaneous squamous cell carcinoma, radiotherapy combined with the anti–PD-1 antibody cemiplimab has shown encouraging clinical activity and acceptable tolerability, suggesting that radiotherapy may be integrated effectively with PD-1 blockade in selected cutaneous malignancies [28]. In this context, higher-dose-rate radiotherapy may be particularly relevant even for larger cutaneous squamous cell carcinoma lesions, as shortening beam-on time could theoretically reduce irradiation of circulating lymphocytes and thereby help preserve systemic immune competence during PD-1 blockade. Although melanoma and cutaneous squamous cell carcinoma are biologically distinct entities, these observations further support the concept of radiotherapy as both a local ablative modality and a potential immunomodulatory partner to checkpoint inhibition.

Importantly, the favorable efficacy observed with combined SBRT and immunotherapy in our cohort was not associated with excess toxicity, consistent with growing evidence supporting the safety of this approach. We did not observe a signal that delivering SBRT during ongoing immunotherapy increased immune-related adverse events, consistent with prospective and retrospective series showing no clear amplification of high-grade toxicity with SBRT–ICI combinations compared with immunotherapy alone [15,29].

This study has several limitations. First, its retrospective, single-center design introduces potential selection bias, limits causal inference, and may reflect institutional treatment practices. Second, the relatively small sample size, particularly in subgroup analyses, reduces the power to detect meaningful differences. Third, the cohort consisted of patients selected for both immunotherapy and metastasis-directed SBRT, with good performance status, limited metastatic burden, and an estimated life expectancy of at least six months. Therefore, these patients likely represent a clinically and biologically favorable subgroup, which may limit generalizability to less selected populations. In addition, a contemporaneous immunotherapy-only control group was not available; thus, this study cannot determine whether SBRT provides a survival advantage over systemic therapy alone. Accordingly, our findings should be interpreted as hypothesis-generating and supportive of prospective evaluation, particularly regarding patient selection and the optimal timing of SBRT relative to immunotherapy.

## 5. Conclusions

In conclusion, our findings suggest that SBRT combined with immunotherapy constitutes a feasible and well-tolerated metastasis-directed treatment strategy for meticulously selected patients with metastatic melanoma. This approach was associated with sustained local control and promising survival outcomes, accompanied by minimal high-grade toxicity. Although numerical differences in treatment sequencing, dose fractionation, and oligometastatic subcategories were observed, these findings did not reach statistical significance and should be regarded as exploratory and hypothesis-generating. Beyond its ablative capacity, SBRT may serve as a biologically complementary partner to immune checkpoint inhibition; however, the clinical significance of this interaction remains to be substantiated. Collectively, these results support prospective studies to enhance patient selection, optimize treatment sequencing, and integrate SBRT with immunotherapy in the management of oligometastatic melanoma.

## Figures and Tables

**Table 1:** Baseline Clinicopathological and Treatment Characteristics of the Study Cohort.

Clinical Variable	n	%
**Sex**		
Male	34	54.0
Female	29	46.0
**Location of the primary melanoma**		
Head and neck	16	25.4
Trunk	25	39.7
Extremities	22	34.9
**Histological subtype of melanoma**		
Superficial spreading melanoma	32	50.8
Nodular	31	49.2
**BRAF status**		
Negative	42	66.7
Positive	21	33.3
**Clinical stage (AJCC 8th edition)**		
IB	6	9.5
IIA	10	15.9
IIB	6	9.5
IIC	4	6.3
IIIA	3	4.8
IIIB	4	6.3
IIIC	7	11.1
IIID	1	1.6
IV	22	34.9
**Immunotherapy**		
Pembrolizumab	46	73.0
Nivolumab	15	23.8
Ipilimumab + Nivolumab	2	3.2
**Timing of SBRT relative to immunotherapy**		
Before	6	9.5
During	32	50.8
After	25	39.7
**Location of treated lesions (n = 97)**		
Liver	14	14.4
Lung	36	37.1
Lymph nodes	38	39.2
Muscle	1	1.0
Bones	5	5.2
Adrenal gland	2	2.1
Pancreas	1	1.0

**Table 2:** Treatment-related adverse events by grade.

Adverse Event	Grade 1, n (%)	Grade 2, n (%)	Grade 3, n (%)	Grade 4–5, n (%)
**SBRT-related toxicity**				
Fatigue	4 (6.3)	1 (1.6)	0	0
Local pain or pain flare	2 (3.2)	1 (1.6)	0	0
Nausea or abdominal discomfort	1 (1.6)	1 (1.6)	0	0
Radiation dermatitis/local skin reaction	1 (1.6)	0	0	0
Radiation pneumonitis	0	1 (1.6)	0	0
Rectal bleeding	0	0	1 (1.6)	0
Total SBRT-related toxicity	8 (12.9)	4 (6.4)	1 (1.6)	0
**Immune-related adverse events after SBRT**				
Skin toxicity	2 (3.2)	1 (1.6)	0	0
Thyroid dysfunction	1 (1.6)	1 (1.6)	0	0
Colitis	1 (1.6)	1 (1.6)	0	0
Arthralgia	1 (1.6)	0	0	0
Transaminase elevation	0	1 (1.6)	0	0
**Total immune-related adverse events after SBRT**	5 (7.9)	3 (4.8)	0	0

Abbreviations: SBRT, stereotactic body radiotherapy. Percentages were calculated using the total number of patients as the denominator (n = 63).

## Data Availability

The original contributions presented in this study are included in the article. Further inquiries can be directed to the corresponding author.

## Abbreviations

The following abbreviations are used in this manuscript:

AJCC	American Joint Committee on Cancer
CT	Computed Tomography
CTCAE	Common Terminology Criteria for Adverse Events
CTLA-4	Cytotoxic T-Lymphocyte-Associated Antigen 4
ECOG	Eastern Cooperative Oncology Group
ICIs	Immune Checkpoint Inhibitors
LC	Local Control
MRI	Magnetic Resonance Imaging
MM	Metastatic Melanoma
MITF	Microphthalmia-Associated Transcription Factor
OS	Overall Survival
PD-1	Programmed Cell Death Protein 1
PD-L1	Programmed Death-Ligand 1
PET/CT	Positron Emission Tomography/Computed Tomography
PFS	Progression-Free Survival
SBRT	Stereotactic Body Radiotherapy
TILs	Tumor-Infiltrating Lymphocytes
